# FcRn Overexpression in Transgenic Mice Results in Augmented APC Activity and Robust Immune Response with Increased Diversity of Induced Antibodies

**DOI:** 10.1371/journal.pone.0036286

**Published:** 2012-04-30

**Authors:** Attila Végh, Anita Farkas, Dorottya Kövesdi, Krisztián Papp, Judit Cervenak, Zita Schneider, Balázs Bender, László Hiripi, Glória László, József Prechl, János Matkó, Imre Kacskovics

**Affiliations:** 1 ImmunoGenes Kft, Budakeszi, Hungary; 2 Department of Immunology, Eötvös Loránd University, Budapest, Hungary; 3 Immunology Research Group, Hungarian Academy of Sciences, Eötvös Loránd University, Budapest, Hungary; 4 Agricultural Biotechnology Center, Gödöllő, Hungary; Federal University of São Paulo, Brazil

## Abstract

Our previous studies have shown that overexpression of bovine FcRn (bFcRn) in transgenic (Tg) mice leads to an increase in the humoral immune response, characterized by larger numbers of Ag-specific B cells and other immune cells in secondary lymphoid organs and higher levels of circulating Ag-specific antibodies (Abs). To gain additional insights into the mechanisms underlying this increase in humoral immune response, we further characterized the bFcRn Tg mice. Our Western blot analysis showed strong expression of the bFcRn transgene in peritoneal macrophages and bone marrow derived dendritic cells; and a quantitative PCR analysis demonstrated that the expression ratios of the bFcRn to mFcRn were 2.6- and 10-fold in these cells, respectively. We also found that overexpression of bFcRn enhances the phagocytosis of Ag-IgG immune complexes (ICs) by both macrophages and dendritic cells and significantly improves Ag presentation by dendritic cells. Finally, we determined that immunized bFcRn mice produce a much greater diversity of Ag-specific IgM, whereas only the levels, but not the diversity, of IgG is increased by overexpression of bFcRn. We suggest that the increase in diversity of IgG in Tg mice is prevented by a selective bias towards immunodominant epitopes of ovalbumin, which was used in this study as a model antigen. These results are also in line with our previous reports describing a substantial increase in the levels of Ag-specific IgG in FcRn Tg mice immunized with Ags that are weakly immunogenic and, therefore, not affected by immunodominance.

## Introduction

The production of monoclonal antibodies (mAbs) using hybridoma technology has allowed significant advances in biomedical research and has greatly improved our capacity for clinical diagnostics and therapeutics. Currently, more than 25 immunoglobulins have been approved for therapeutical use in humans and over 240 antibodies are in development targeting a wide variety of diseases, including autoimmunity, cancer, infectious diseases and cardiovascular diseases (reviewed by [1]).

In recent years, there has been an increasing demand for the development of cheaper, faster and more efficient technologies for the production of high-affinity and high-specificity mAbs. One approach to improve the efficiency of hybridoma production is to enhance humoral immune response against various antigens (Ags), including weakly immunogenic targets to which mAbs are generally difficult to generate. Another approach is to create a higher diversity of Ag-specific antibodies, allowing for the development of a larger variety of hybridomas, which can be screened for their ability to bind native epitopes and to produce functionally relevant mAbs [2]. To achieve these goals, our group has recently created transgenic (Tg) mice that overexpress the bovine neonatal Fc receptor (bFcRn) [3] and show a greatly augmented humoral immune response. Our previous analyses have shown that the bFcRn Tg mice offer major advantages for hybridoma production and could serve as important tools for the development of new therapeutic mAbs [4]. In addition, we have recently generated Tg rabbits that overexpress the rabbit FcRn and observed similarly improved IgG protection and enhanced humoral immune response as described for bFcRn Tg mice [5].

The neonatal Fc receptor (FcRn) is a MHC Class I-related receptor composed of an α-chain and β2-microlobulin (β2m) [6] and was originally identified as the protein that mediates the transport of IgG from maternal milk to the small intestine of newborn rodents [7]. FcRn has proven to be a key player in regulating the transport of IgG within and across cells of diverse origins and it also serves to rescue IgG and albumin from degradation, thereby prolonging their half-lives [8]. IgG protection was originally thought to be mediated by capillary endothelial cells [9] but recent findings suggest that this process also occurs in hematopoietic cells [10], [11] and in mammary epithelial cells during lactation [12]. More recently, several publications have shown that FcRn plays major roles in Ag-IgG immune complex (IC) phagocytosis by neutrophils [13], and in Ag presentation of IgG ICs by professional Ag presenting cells (APCs) [14], [15], [16], [17].

We have recently shown that overexpression of bFcRn in Tg mice leads to increased levels of IgG in the serum as a result of a reduction in IgG catabolism. In addition, we found that expression of bFcRn in Tg mice causes an increase in the levels of Ag-specific IgG and IgM during the secondary immune response and leads to an enhanced expansion of Ag-specific B cells and plasma cells in their spleen [18], [19]. We also observed that, upon immunization, bFcRn Tg mice develop enlarged spleens that contain higher numbers of neutrophil granulocytes and dendritic cells (DCs) as compared to wild-type (wt) mice [18], [20]. This augmented immune response is also reflected in the ability of bFcRn Tg mice to produce high levels of Ag-specific antibodies, B cells and plasma cells to weakly immunogenic targets [20] and to produce elevated numbers of Ag-specific hybridomas [19].

To better understand the mechanisms underlying the augmented humoral immune response observed in bFcRn Tg mice, we further characterized the profile of bFcRn transgene expression in different cells of the immune system. In addition, we investigated the effects of overexpression of bFcRn in the phagocytic activity of macrophages and dendritic cells, as well as its effect on Ag-presentation by dendritic cells. Moreover, we investigated whether the enhanced immune response in bFcRn Tg mice correlates with an increase in the diversity of Ag-specific immunoglobulins.

## Materials and Methods

### Ethics statement

All the treatments of animals (mice) in this research followed the guidelines of the Institutional Animal Care and Ethics Committee at Eötvös Loránd University that operated in accordance with permissions 22.1/828/003/2007 issued by the Central Agricultural Office, Hungary and all animal work was approved by the appropriate committee.

### Mice

We used hemizygous transgenic mice that carry five copies of the bovine FcRn α-chain encoding gene (bovine FCGRT) in addition to the endogenous mouse FCGRT gene on BALB/c genetic background (BALB/c_Tg5_bFCGRT(19); 19 refers to the founder line) we have previously generated [18]. As controls, we used wt littermates and standard BALB/c mice. Tg and wt mice were used for peritoneal macrophage isolation (10-week old), bone marrow derived dendritic cell generation (4–8 week old), cell-specific bFcRn detection and epitope mapping (female mice; 8–10 week old). For T cell proliferation assays, we used mice carrying the MHC class II restricted rearranged T cell receptor transgene Tg(DO11.10)10Dlo, which binds to ovalbumin (OVA) peptide antigen (OVA 323–339 peptide) purchased from The Jackson Laboratory (Bar Harbor, ME, USA).

### Cells

#### Isolation of splenic B-cells, T-cells

Isolation of splenic B-cells, T-cells splenocytes were incubated with fluorochrome-conjugated specific Abs at 4°C for 50 min in PBS and subjected to cell sorting using FACSAria III cell sorter (BD Biosciences, Franklin Lakes, NJ, USA) equipped with FACSDiva v6.1.3 software. Phycoerythrin (PE)-labeled anti CD3-(eBioscience, San Diego, CA, USA) and FITC-labeled anti CD19 (eBioscience) were used to isolate T cells and B cells, respectively.

#### Isolation of peritoneal neutrophil granulocytes

Isolation of peritoneal neutrophil granulocytes mice were injected i.p. with 1 ml of 10 mg/ml casein (Sigma-Aldrich, Budapest, Hungary) in sterile saline. The procedure was repeated 12 hours later and peritoneal cells were isolated 3 hours after the second injection. Neutrophils were then further purified by Ficoll-Paque PLUS (GE Healthcare, Uppsala, Sweden) centrifugation (400× *g* for 30 min at RT) and its purity was determined by flow cytometry using Alexa Fluor 647-labeled anti-CD11b (eBioscience) and PE-labeled anti-Gr-1 (BD Pharmingen) reagents.

#### Isolation of peritoneal macrophages

Isolation of peritoneal macrophages mice were injected i.p. with 1 ml of 3% Brewer thioglycolate medium, euthanized 3 days later and the peritoneal exudate cells were harvested. The purity of peritoneal macrophages was determined with Alexa Fluor 647-labeled anti-CD11b and PE-labeled anti-Gr1.

#### Differentiation of dendritic cells from bone marrow

Differentiation of dendritic cells from bone marrow bone marrow cells were isolated from femurs and tibias and cultured at 2×10^6^ cells/ml in 6-well cell culture dishes containing 2 ml of RPMI II, 10% FCS, 10 ng/ml rmGM-CSF (Millipore, Billerica, MA, USA) and 10 ng/ml rmIL4 (eBioscience). Two-thirds of the media were replaced every 2 days. After 7 days of treatment, the differentiated dendritic cells were stained by using PE-labeled anti-MHCII (I-A/I-E) (eBioscience), Alexa Fluor 647-labeled anti-CD11b, PE-labeled anti-CD11c (BD Pharmingen), PE-labeled anti-Gr1 (Ly6-G) (BD Pharmingen), and FITC-labeled anti-CD14 (BD Pharmingen) and analyzed by flow cytometry. We also analyzed the expression of the FcγRs with K9.361 [21], MHC class II with M5/114 (TIB120; ATCC, USA) and B7.1 with 1610A [22] and B7.2 with HB-253 (GL1, ATCC) on these cells. Isotype controls were obtained from BD Pharmingen or eBioscience.

### bFcRn detection by Western blot analysis

#### Generation of bFcRn-specific monoclonal antibody

Generation of bFcRn-specific monoclonal antibody 2 month old female BALB/c mice were immunized with 50 µg soluble bFcRn (sbFcRn) molecule [23] in CFA intraperitoneally (i.p.) and boosted two times with 3 week intervals with 50 µg sbFcRn in IFA i.p. On day 63, 25 µg sbFcRn was given intravenously and the same amount i.p. Fusion and hybridoma selection were performed under the same conditions as described earlier [19]. Hybridoma microculture supernatants were first screened in a sbFcRn specific ELISA assay; then the positive clones were further tested in Western blots and finally a positive microculture was selected for cloning using limiting dilution method, resulting in a bFcRn-specific clone (1E5/2).

#### Western blot

Spleen, splenic B-cells, T-cells, peritoneal neutrophil granulocytes, peritoneal macrophages and bone marrow derived dendritic cells from bFcRn Tg mice as well as spleen from control (BALB/c) mice were lysed, and their protein concentrations were determined by Micro BCA Protein Assay kit (Pierce Biotechnology, Rockford, IL, USA). 20 and 5 µg of peritoneal macrophages and bone marrow derived dendritic cells and 20 µg of spleen, B cells, T cells and peritoneal neutrophil granulocytes were subjected to electrophoresis on 10% SDS-PAGE gel and transferred to Immobilon P PVDF membrane (Millipore, Billerica, MA, USA). Blot was probed with the supernatant of our mouse anti-bFcRn specific monoclonal antibody (1E5/2; conc: 26 µg/ml) according to standard protocol. bFcRn α-chain protein was detected with horseradish peroxidase-conjugated goat anti-mouse IgG antibody (Southern Biotech, Birmingham, AL, USA) and enhanced chemiluminescence, using SuperSignal West Pico Chemiluminescent Substrate (Thermo Scientific, Rockford, IL, USA). bFcRn stable-transfected bovine mammary epithelial cell line B4 [23] was used as positive control. The same blot was rehybridized with anti-β-actin monoclonal antibody as loading control.

### bFcRn and mouse FcRn (mFcRn) α-chain gene expression in spleen, peritoneal macrophages, and bone marrow derived dendritic cells

Total RNA was extracted by using RNeasy® Plus Mini Kit (that includes a DNase digestion step, Qiagen GmbH, Germany) from spleen, peritoneal macrophages, and bone marrow derived dendritic cells of transgenic mice and first strand of cDNA was synthesized using the High Capacity cDNA Reverse Transcription Kit (Life Technologies, USA). Quantitative PCR was run with RotorGene RG-3000 (RotorGene software, Corbett Research, Sidney, Australia). Relative quantifications with efficiency correction of the individual transcripts were determined according to the mathematical model described earlier [24]. PrimeTime double quenched assays (Integrated DNA Technologies, Coralville, Iowa, IA, USA) using primer and probe sets for bFcRn, mFcRn and mouse polyubiquitin-B (to normalize qRT-PCR data) (**Table 1**) were performed. Samples contained 100 ng DNA per PCR reaction which was initiated with a denaturation step at 95°C 10 min, followed by 40 cycles of 95°C 15 sec, 60°C 60 sec.

**Table 1:** Sequences of PrimeTime PCR primer/probe sets used to measure the expression of bFcRn, mFcRn and mouse poliubiquitin.

Target	Reference sequence in GenBank	Primer and probe sequences
bFcRn	NM_176657	F-5′-TCTCCTTCTACCCACCTGAG-3′
		R-5′-GTCTATCTCACCAGAGCCAATG-3′
		P-5′/56-FAM/CAGCCCGTT/ZEN/CCGCAGAAAGC/3IABkFQ/-3′
mFcRn	NM_010189	F-5′-CGTTCTTGGTTTATTGCTGGTG-3′
		R-5′-CAACAGGTCACCAGAGTCATC-3′
		P-/56-FAM/CCCCATGGC/ZEN/TTTCTCTCAGCGG/3IAkFQ/-3′
mouse polyubiquitin B (Ubb)	NM_011664.3	F-5′-TCTTTCTGTGAGGGTGTTTCG-3′
		R-5′-GTTCTCGATGGTGTCACTGG-3′
		P-5′-/56-FAM/CTAGGGTGA/ZEN/TGGTCTTGCCGGTC/3IABkFQ/-3′

### Confocal microscopy

For confocal imaging we used an Olympus Fluoview 500 confocal microscope (Hamburg Germany) equipped with four optical channels and a 60× (numerical aperture of 1.45) oil immersion objective.

### Phagocytosis assay

For the phagocytosis assays, we used an Alexa Fluor 488-labeled IC that contains Alexa Fluor 488-labeled OVA and anti-OVA IgG. The anti-ovalbumin IgG was purified from sera of OVA immunized mice. 10 µg/well of IC or soluble OVA were added to 5×10^5^ bone marrow-derived DCs and 5×10^5^ peritoneal macrophages. Cells were incubated at 37°C and 5% CO_2_ for 30, 60, 90, and 120 min. As controls, cells were incubated with either IC or OVA on ice. After phagocytosis, cells were washed at 4°C and the bone marrow-derived DCs were labeled with PE-labeled anti-MHCII (I-A/I-E), and Alexa Fluor 647-labeled anti-CD11b, while the peritoneal macrophages were labeled with Alexa Fluor 647-labeled anti-CD11b and PE-labeled anti-Gr1 (Ly6-G). Samples were analyzed by flow cytometry and confocal microscopy.

### T-cell proliferation assay

Bone marrow-derived DCs were added to 96-well plates (2×10^5^ DC/well) and were left untreated or incubated with either IC or soluble OVA (100 µg/ml) for 1 hour at 37°C and 5% CO_2_. Mouse CD4^+^ T cells from DO11.10 mice bearing an OVA-reactive transgenic TCR were purified by negative selection with CD8^+^ and CD19^+^ microbeads (Miltenyi Biotec, Bergisch Gladbach, Germany). Purified CD4^+^ T cells (4×10^5^ cells/well) were then added to the untreated, IC- or OVA–treated bone marrow-derived DCs. As a positive control, T cells were treated with 1 µg/ml of Concanavalin A (ConA) (Sigma-Aldrich, St. Louis, MO, USA). Cells were incubated at 37°C, 5% CO_2_ for 24 hours and then labeled with 37 kBq [H3]-thymidine for 12 hours. T cell proliferation was determined by [H3]-thymidine incorporation.

### Ovalbumin (OVA) immunizations and ELISA

15 BALB/c_Tg5_bFCGRT (19) and 15 wt control mice were immunized i.p. with 200 µg OVA in Complete Freund Adjuvant (CFA) and challenged i.p. with 50 µg OVA in Incomplete Freund Adjuvant (IFA) on days 21 and 42. Animals were bled on days 0, 21, 28, 42 and 49; sera were harvested and stored in aliquots at −20°C until use. Ag-specific immunoglobulin (IgG and IgM) titers were measured by ELISA as described before [18].

### Microarray preparation

Based on the sequence of OVA (NCBI Reference Sequence: NP_990483.1), an overlapping library of 95 12-mer peptides was synthesized by JPT Peptide Technologies (Berlin, Germany). Peptides that cover the whole sequence of OVA with an offset of 4 amino acids and an overlap of 8 amino acids were biotinylated on their N-terminus and mixed with streptavidin at a 1∶4 molar concentration (**Figure S1**). Peptide-streptavidin complexes were then printed at a 1 mg/ml concentration (diluted in PBS containing 0.05% Na-azide) onto glass slides covered with a nitrocellulose membrane in a two-pad format compatible with Whatman FAST Slide incubation chambers. Mouse IgG, goat anti-mouse IgG (Jackson ImmunoResearch Laboratories, Newmarket, Suffolk, UK), goat anti-mouse IgM (Jackson ImmunoResearch Laboratories), mouse serum, and mouse albumin were printed at different dilutions as internal and interassay controls. Features were printed in triplicates at a density of 100 spots/cm^2^ and with a diameter of approximately 400 µm using the BioOdyssey Calligrapher miniarrayer (BioRad, Hercules, CA, USA). Slides were stored at 4°C in sealed bags until use.

### Testing serum samples

Samples collected on day 49, one week after the last immunization, were tested individually on microarrays. Pooled samples were prepared on days 21, 28, 42 and 49 and tested to determine the dynamics of the humoral immune response of the animals. Slides were put into Whatman FAST Slide incubation chambers and washed 3× for 10 min with PBS on an orbital shaker. The slides were then blocked with dilution buffer (PBS containing 25 mM EDTA, 5% BSA, and 0.05% Tween-20) for 1 hour and washed with PBS-Tween for 10 min. Slides were then incubated with sera diluted in dilution buffer (1∶25 for IgM and 1∶2500 for IgG measurements) at 37°C for 1 hour, washed twice for 10 min with PBS-Tween and pulled out of the incubation chambers. 7 ml of diluted goat Cy5-labeled anti-mouse IgG (SouthernBiotech, Birmingham, AL, USA) (1∶5000) or FITC-labeled rat anti-mouse IgM (eBioscience) (1∶2500) were added for 30 minutes at RT. Slides were washed twice for 10 min with PBS-Tween and spun dry.

### Scanning and data analysis

Microarrays were scanned with the Axon GenePix 4200A (Molecular Devices, Sunnyvale, CA, USA) equipped with GenePix Pro 7.0 (Molecular Devices, Corp.) software on 25% power, 530 PMT gain and 20 µm pixel size. Fluorescence intensity was calculated for each spot as the median fluorescence of the feature minus the fluorescence of the local background. The signal was considered positive when the fluorescence intensity of the spot was higher than the average fluorescence intensity of printed streptavidin. For interassay comparison, data were normalized to the mean fluorescence intensity of control samples, which consist of mouse IgG in the case of IgG measurements and total mouse serum for IgM measurements.

## Results

### bFcRn α-chain is strongly expressed in peritoneal macrophages and bone marrow derived dendritic cells of the bFcRn Tg mouse

Our previous studies of the bFcRn Tg mice revealed that overexpression of the bFcRn leads to a general increase in secondary humoral immune response, in addition to the increase in serum IgG levels. To further understand this effect, we have investigated the expression profile of the bFcRn transgene in splenic B cells and T cells, as well as in peritoneal macrophages, bone marrow derived dendritic cells and peritoneal neutrophil granulocytes at protein and mRNA levels.

#### Expression at protein level

**Figure 1:**
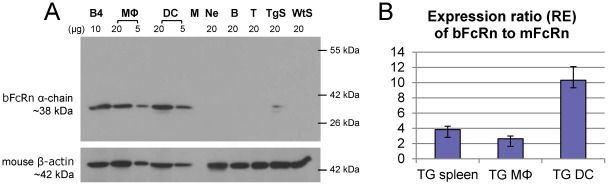
bFcRn is strongly expressed in bone marrow derived dendritic cells and peritoneal macrophages of bFcRn Tg mice. **A.** Western blot analysis shows the expression of bovine α-chain protein in spleen (TgS), B cells (B, 99.4% purity), T cells (T; 99.5% purity), peritoneal neutrophil granulocytes (Ne; 91.4% purity), bone marrow derived dendritic cells (DC; 97.8% purity), and peritoneal macrophages (MΦ; 99.3% purity) from non-immunized bFcRn Tg animals. Based on its molecular weight, the 38-kDa band was specific for bFcRn as also evidenced by its expression in the bFcRn stable transfected cells (B4) [23] for positive and spleen (WtS) from a control BALB/c mouse as negative control, respectively (which also indicates that our bFcRn specific 1E5/2 mAb does not cross react with mFcRn α-chain). Blot was stripped and rehybridized with an anti-β-actin monoclonal antibody as a loading control. **B.** Expression ratios of the bFcRn to mFcRn at mRNA level in peritoneal macrophages (MΦ; 99.3% purity), bone marrow derived dendritic cells (DC; 97.8% purity) and spleen from bFcRn Tg mice analyzed with a quantitative real-time PCR assay. We found that the bFcRn expression was 3.8-, 2.6- and 10-fold higher compared to the expression of the mFcRn in spleen, macrophages and dendritic cells, respectively. Results are representative of 3 independent experiments and show as the mean ±SD.

Expression at protein level spleen, B cells (99.4% purity), T cells (99.5% purity), peritoneal neutrophil granulocytes (91.4% purity), bone marrow derived dendritic cells (97.8% purity), and peritoneal macrophages (99.3% purity) were isolated from non-immunized bFcRn Tg animals. The expression of bovine α-chain protein was analyzed by Western blot using our newly developed monoclonal antibody 1E5/2. Based on its molecular weight, the 38-kDa band was specific for bFcRn as also evidenced by its expression in the bFcRn stable transfected cells (B4) [23] for positive and the spleen sample from a control BALB/c mouse as negative control, respectively (which also indicated that our 1E5/2 mAb does not cross react with mFcRn α-chain). Blot was stripped and rehybridized with an anti-β-actin monoclonal antibody as a loading control. We detected strong bFcRn α-chain protein expression in macrophages and dendritic cells of bFcRn Tg mouse. Tg spleen showed α-chain protein expression, too, however its expressional level was much lower compared to the macrophages and dendritic cells. Since the bFcRn expressional level was undetectable in B-cells and T-cells that dominates the non-immunized spleen or in neutrophil granulocytes, the weak bFcRn expression in spleen probably reflects bFcRn expression in the splenic macrophages and dendritic cells (**Figure 1A**).

#### Expression at mRNA level

Expression at mRNA level we also analyzed the expression ratios of the bFcRn to mFcRn at mRNA level in peritoneal macrophages (99.3% purity), bone marrow derived dendritic cells (97.8% purity) and spleen from bFcRn Tg mice with a quantitative real-time PCR assay. We found that the bFcRn expression was 3.8-, 2.6- and 10-fold higher compared to the expression of the mFcRn in spleen, macrophages and dendritic cells, respectively (**Figure 1B**). This dataset indicates a significantly higher level expression of the bFcRn than its mouse counterpart due to the fact that these Tg mice carry and efficiently express 5 copies of the bFcRn transgene. The more robust bFcRn expression in the bone marrow derived dendritic cells is probably the result of the cytokine stimulus (IL-4, GM-CSF) used to generate these cells.

### FcRn overexpression leads to an increase in OVA-IgG immune-complex phagocytosis

**Figure 2:**
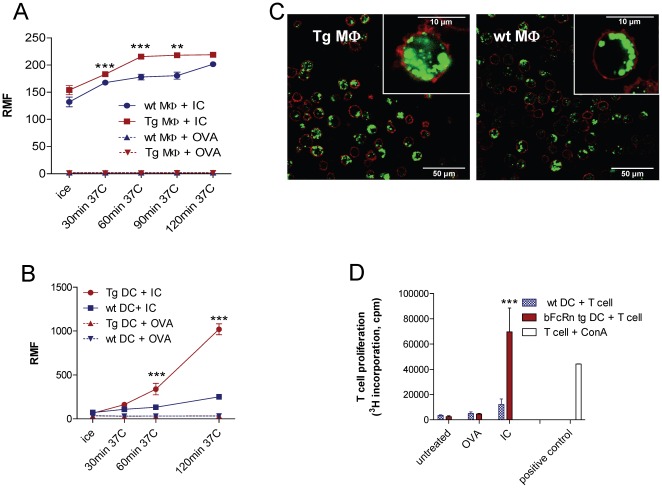
bFcRn-expressing cells show enhanced IC phagocytosis and induce higher T cell proliferation. (**A**) Peritoneal macrophages (MΦ) and (**B**) bone marrow-derived dendritic cells (DC) isolated from bFcRn Tg and wt mice were incubated with Alexa Fluor 488-conjugated OVA-IgG immune complex (IC) or with Alexa Fluor 488-conjugated OVA (OVA) alone at 37°C for the indicated times. As negative controls, cells were left on ice for 30 minutes in the presence of either IC or OVA. The uptake was analyzed by flow cytometry using anti-CD11b-Alexa Fluor 647 and anti-Gr1 (Ly6-G)-PE as markers for macrophages, and anti-MHCII (I-A/I-E)-PE and anti-CD11b-Alexa Fluor 647 for dendritic cells. The results are expressed in RMF (relative mean fluorescence). Values shown are the means ± SD (*, *P*<0.05; **, *P*<0.01; ***, *P*<0.001). The experiments were repeated three times, with three parallels with similar results. (**C**) Peritoneal macrophages (MΦ) from bFcRn Tg and wt mice were incubated with Alexa Fluor 488 conjugated OVA-IgG (green) at 37°C for 60 min. Cells were then washed, labeled with Alexa Fluor 647-conjugated anti-CD11b (red) and visualized by confocal microscopy. (**D**) Bone marrow-derived dendritic cells (DC) from wt and bFcRn Tg mice were left untreated or loaded with either OVA-IgG immune complex (IC) or OVA alone for 1 hour at 37°C and 5% CO_2_. CD4+ T cells from OVA TCR (DO11.10) Tg mice were then added to both the untreated and loaded DCs. After 24 hours, proliferating T cells were labeled for 12 h with [H3]-thymidine. As a positive control, CD4+ T cells were incubated with Concavalin A (ConA). Results are representative of 3 independent experiments and show as the mean ±SD (*, *P*<0.05; **, *P*<0.01; ***, *P*<0.001).

We next investigated the effects of FcRn overexpression in the phagocytic activity of APCs. We isolated peritoneal macrophages and differentiated bone marrow-derived dendritic cells from bFcRn Tg and wt mice and performed an in vitro phagocytosis assay using an Alexa Fluor 488-labeled OVA-IgG immune complex (IC) or Alexa Fluor 488-labeled OVA alone. We found that both the peritoneal macrophages (CD11b^+^Gr1^−^) and the bone marrow DCs (MHCII^+^CD11c^+^CD11b^+^) derived from bFcRn Tg mice have significantly higher phagocytic activity than the corresponding cells from wt mice (**Figure 2A and B**). We confirmed the intracellular localization of the phagocytosed ICs by confocal microscopy (**Figure 2C**). We have not detected any difference in the cell surface expression of the FcγRs, MHC class II and B7.1, B7.2 molecules on the Tg and wt dendritic cells (data not shown).

### Bone marrow-derived DCs from bFcRn Tg mice have enhanced Ag presentation ability

To determine whether the increase in phagocytic activity of bone marrow-derived DCs correlates with a higher Ag presenting ability, we performed an Ag-specific T-cell proliferation assay. We found that bone marrow-derived DCs from bFcRn Tg mice loaded with OVA-IgG IC induce significantly higher CD4^+^ T-cell proliferation as compared to bone marrow-derived DCs from wt mice (**Figure 2D**). No differences were observed in the levels of stimulation of T cell proliferation by Tg and wt bone marrow-derived DCs loaded with soluble OVA alone (**Figure 2D**).

### More robust increase of specific IgM and IgG antibodies over time in Tg mice

**Figure 3:**
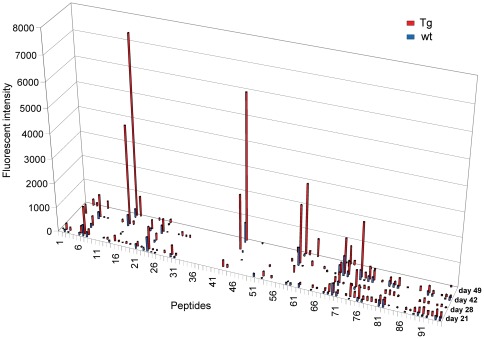
bFcRn Tg mice show higher levels and increased diversity of peptide-specific IgM at 21, 28, 42 and 49 days after initial OVA immunization. Sera from 15 bFcRn Tg (red) or 15 wt (blue) mice immunized with OVA were collected on the indicated days, pooled and tested on OVA overlapping peptide microarrays. IgMs bound to different peptides were detected with rat anti-mouse IgM conjugated to FITC and the fluorescence intensity was calculated for each spot as the median fluorescence of the spot minus the fluorescence of the local background. Numbers on the X-axis refer to the specific OVA peptide on the microarray slide.

**Figure 4:**
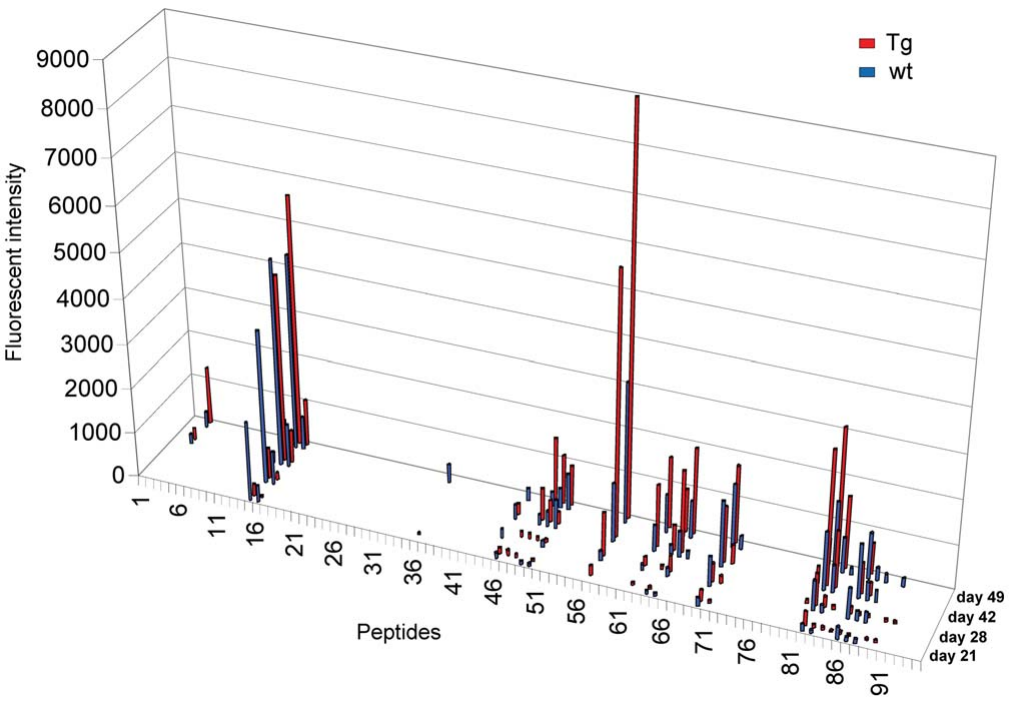
bFcRn Tg mice show increased levels of epitope-specific IgGs. Sera from 15 bFcRn Tg (red) or 15 wt (blue) mice immunized with OVA were collected on the indicated days, pooled and tested on OVA overlapping peptide microarrays. IgGs bound to different peptides were detected with a goat anti-mouse IgG conjugated to Cy5 and the fluorescence intensity was calculated for each spot as the median fluorescence of the spot minus the fluorescence of the local background. Numbers on the X axis refer to the specific OVA peptide on the microarray slide.

As previously reported, bFcRn Tg mice produce three times more OVA-specific IgG than wt mice ([18] and data not shown) and the augmented antigen-specific humoral immune response in Tg mice results in larger numbers of antigen specific B cells [18], [19], [20]. To investigate whether the higher levels of antibody in bFcRn Tg mice correlate with an increase in the diversity of recognized peptides, we used microarrays containing a library of overlapping peptides that cover the whole sequence of OVA. These microarrays allow for the identification of linear epitopes recognized by antibodies present in the serum of immunized mice. First, we investigated the effects of FcRn overexpression on the overall dynamics of the humoral immune response. Sera from 15 bFcRn Tg (red) and 15 wt mice (blue) were collected on days 21, 28, 42 and 49 after immunization, pooled, and tested on the OVA peptide microarrays. On day 21 (secondary immune response), both wt and bFcRn Tg mice produced IgM that recognized several peptides in the microarrays, albeit with low signal intensity. In wt mice, there was little change in the levels of peptide-specific IgM throughout the entire immunization protocol. In contrast, the Tg mice produced continuously increasing amounts of epitope-specific IgM Abs, which in several cases, reached their highest signal intensity on day 49 (**Figure 3**). The amount of peptide-specific IgGs increased over time in both groups, with a more robust increase in Tg mice in some peptides (**Figure 4**).

### bFcRn Tg mice show increased levels and higher diversity of IgM than wt mice

**Figure 5:**
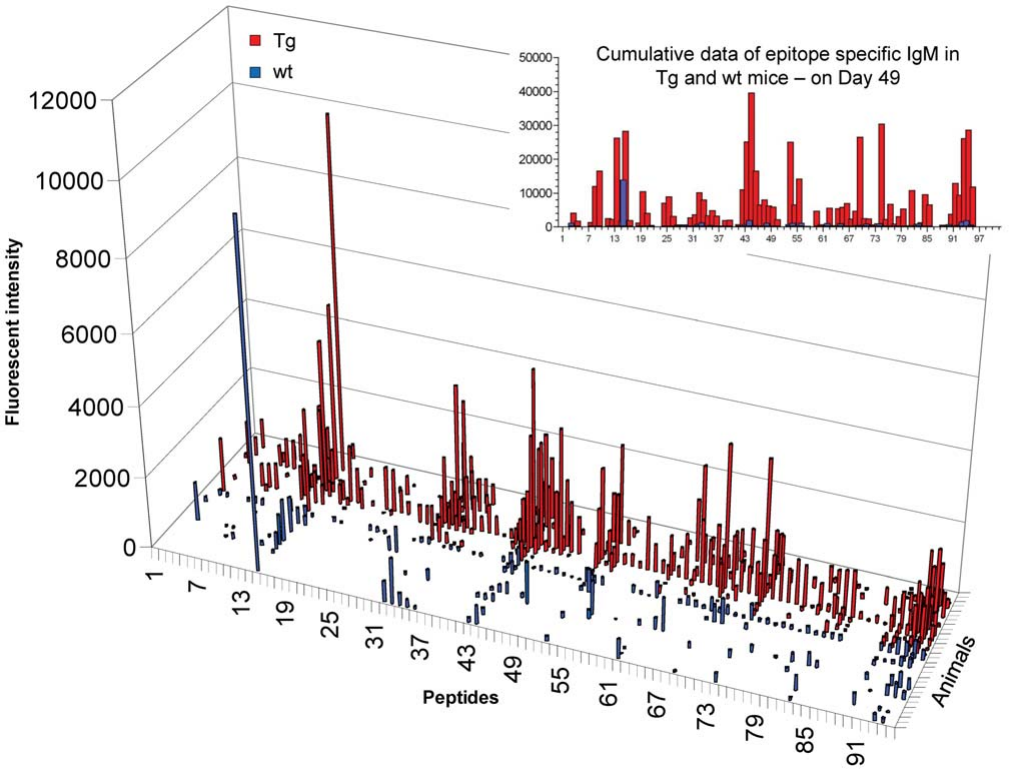
bFcRn Tg mice show increased levels and higher diversity of epitope-specific IgM as compared to wt mice. Sera from 15 bFcRn Tg (red) and 15 wt (blue) mice immunized with OVA were collected on day 49 and tested individually on microarrays containing 95 overlapping 12-mer peptides that cover the whole sequence of ovalbumin. IgMs bound to different peptides were detected with a rat anti-mouse IgM conjugated to FITC and the fluorescence intensity was calculated for each spot as the median fluorescence of the spot minus the fluorescence of the local background. Numbers on the X axis refer to the specific OVA peptide in the microarray. For comparison between assays, the data were normalized to the mean fluorescence obtained for whole serum of a naïve mouse. The cumulative data represents the addition of all the fluorescence intensity values obtained for each peptide-specific IgG from bFcRn Tg or wt mice on day 49.

**Figure 6:**
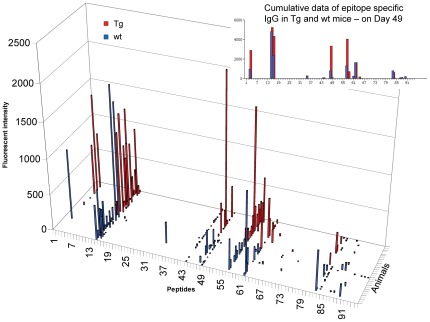
bFcRn Tg mice show higher levels of epitope-specific IgGs as compared to wt mice. Sera from 15 bFcRn Tg (red) and 15 wt (blue) mice immunized with OVA were collected on day 49 and tested individually on microarrays containing 95 overlapping 12-mer peptides that cover the whole sequence of ovalbumin. IgGs bound to different peptides were detected with a goat anti-mouse IgG conjugated to Cy5 and the fluorescence intensity was calculated for each spot as the median fluorescence of the spot minus the fluorescence of the local background. Numbers on the X axis refer to the OVA peptide in the microarray. For comparison between assays, the data were normalized to the mean fluorescence of purified mouse IgG. The cumulative data represents the addition of all the individual fluorescence intensity values obtained for each peptide-specific IgG from either bFcRn Tg or wt mice.

To investigate the individual variation within the group of Tg or wt mice, serum collected from each mouse on day 49 was individually tested on OVA peptide microarrays. First, we examined whether overexpression of bFcRn affects the diversity of IgM. We performed epitope mapping of IgM from sera collected on day 49 and observed clear differences between the groups of Tg and wt mice. All of the individual Tg mice displayed significantly higher levels and diversity of IgM as compared to wt mice (**Figure 5**). We observed that all the peptides recognized by IgG (**Figure 6**) were also recognized by IgM. A larger number of individuals from the bFcRn Tg group produced IgM against a given peptide and, consequently, the cumulative signal intensity for each peptide was much higher for the Tg animals as compared to the wt controls (**Figure 5** **)**.

**Figure 7:**
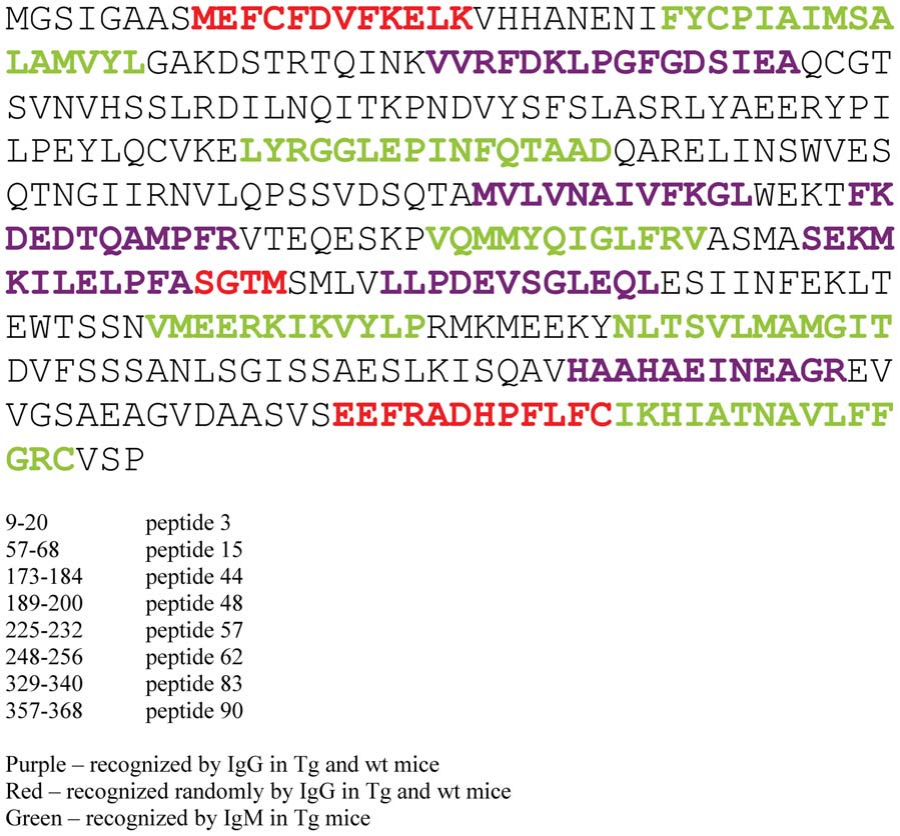
Amino acid sequence of OVA; epitopes recognized by IgG and IgM of bFcRn Tg and wt mice. Epitopes marked in purple are recognized by IgG from both bFcRn Tg and wt mice. Epitopes marked in red are recognized by IgG of either bFcRn Tg or wt mice, and the epitopes marked in green are recognized by IgM from bFcRn Tg mice. (In case of peptide 58, only the non-overlapping amino acids are indicated with red color.)

Several peptides that were not recognized by IgG from neither Tg nor wt mice were positive for IgM produced by bFcRn Tg mice (**Figure 7**). The bFcRn Tg mice showed significantly higher diversity and levels of IgM, displaying positive signals to several peptides (e.g., 32, 33, 44 53, 69, 74, 93 and 94), while the wt mice showed minimal or no signal for these peptides (**Figure 5** **and** **7**). Analyzes of individual samples revealed the diversity of peptide-specific IgM Abs, which was not seen in the pooled sera (**Figure 3**).

### bFcRn Tg mice produce higher levels of epitope-specific IgGs than wt mice

Next, we analyzed whether overexpression of bFcRn affects the diversity of IgG. The number of animals that show a positive signal for a given peptide was highly variable in both groups. Almost all animals produced IgG that recognized peptides 15 and 57, while approximately half of the animals from each group had IgG to peptides 16, 48 and 62. Some OVA peptides were only recognized by IgG from a small number of Tg and wt mice (**Figure 6**). Cumulative signal intensities were calculated for each peptide to determine the overall antibody production against specific epitopes in the two groups. Data show that the majority of peptides recognized in both groups have higher cumulative intensity in the Tg group (**Figure 6**).

## Discussion

Over the last 35 years, mAbs have become an essential tool for clinical research and have been increasingly used for the diagnosis and treatment of malignant, inflammatory, autoimmune and infectious diseases. Despite the recent development of alternative methods to generate recombinant antibodies, such as in vitro display technologies, murine hybridoma technology is still the most widely employed method for producing mAbs. In order to screen and produce effective antibodies with high affinity and specificity, numerous hybridoma clones need to be generated, especially to generate functionally relevant mAbs that bind native epitopes and can be used for therapeutic approaches [2]. However, some challenges remain to improve the efficiency of clone generation. For example, the number of B cells secreting a specific antibody can be as low as 1% of the total number of lymphocytes in an immunized mouse. The efficiency of fusion of splenocytes and myeloma cells is also very low and only a small number of fused cells are able to grow as hybridomas during selection [25]. Finally, the stability of the generated clones is also uncertain and a large fraction of hybridomas stop secreting over time, due to random shedding of chromosomes until they reach a stable genomic configuration [26].

Our group has recently generated Tg mice overexpressing the bFcRn gene, which show a significant increase in the levels of serum IgG and IgM in response to a variety of Ags without presenting any signs of autoimmunity [3], [18], [19], [20]. We also observed that the bFcRn Tg mice develop enlarged spleens containing increased numbers of neutrophil granulocytes, dendritic cells, Ag-specific B cells and plasma cells, which allow for a significantly improved efficiency of hybridoma production [19]. In parallel with this study, we have recently tested whether the bFcRn Tg mice would yield specific antibodies against a clinically relevant, yet weakly immunogenic target. In that study, we immunized both Tg and wt mice with the human chemokine receptor CXCR4, which belongs to the G protein-coupled receptor (GPCR) family and plays important roles in the metastatic spread of cancer cells as well as in the entry of HIV in CD4+ cells [27], [28]. Using cells transfected with CXCR4 as the immunogen, we were able to isolate four anti-CXCR4 positive hybridoma clones from bFcRn Tg mice whereas the BALB/c wt animals did not produce a single CXCR4-specific clone [29]. In addition, one of the four CXCR4-specific mAbs binds a functionally relevant epitope on this receptor as it inhibits the binding of a well characterized CXCR4-specific mAb (12G5) [30].

In our recent publications, we proposed that overexpression of bFcRn leads to a better protection of the Ag-specific IgGs which, in turn, can generate a greater amount of Ag-IgG ICs. The ability of ICs to induce potent humoral immune responses has long been known [31], [32], [33], [34]. We found that immunization significantly increases the number of neutrophil granulocytes in the spleen and that this change is more pronounced in Tg animals than in the wt controls [18], [20]. This is consistent with other recent observations indicating that, in the presence of Ag-IgG ICs, neutrophils are the main Ag-specific cells recruited to draining lymph nodes and that their numbers depend on the levels of ICs [35], [36], [37]. Since the bFcRn Tg mice have higher levels of Ag-specific IgGs than wt mice, we concluded that the difference in the numbers of neutrophils in Tg and wt mice can be explained, at least in part, by the presence of higher levels of Ag-IgG ICs formed and/or transported by neutrophils in Tg animals. Furthermore, it has been shown that FcRn is expressed in neutrophils and plays an active role in Ag-IgG IC phagocytosis [13]. We have recently shown that FcRn overexpression in neutrophils of bFcRn Tg mice results in more efficient phagocytosis [18], which further boosts their activation, and contributes to their increased influx into the draining secondary lymphoid tissues or spleen. The emerging evidence of the important and multifaceted roles of neutrophil granulocytes in potentiating the adaptive immune response in secondary lymphoid organs have been recently reviewed [38].

We also found that immunization leads to a significant increase in the number of dendritic cells in the spleen and that this change was, similarly to that observed for neutrophils, more striking in bFcRn Tg animals as compared to wt mice [18], [20]. Recent studies have shown that FcRn is expressed in APCs [10], [15], [39] and efficiently increases phagocytosis and recycling of monomeric IgG in these cells. In addition, FcRn was found to direct Ag-IgG ICs into lysosomes and to play a role in Ag presentation and humoral immune responses [14], [15], [16], [17]. Taken together, the data indicate that FcRn redirects Ags complexed with IgG into degradative compartments that are associated with the loading of antigenic peptides onto MHC class II molecules within cells [8]. The larger number of dendritic cells observed in bFcRn Tg animals after immunization suggests that these mice have higher Ag-presenting capability than wt mice [18], [20].

In this current study, we also addressed whether the APCs from bFcRn Tg mice, including macrophages, dendritic cells and B cells, express the bFcRn transgene and if they do, how this expression affects their phagocytic and Ag presentation abilities. Our results indicate that the elevated immune response observed in bFcRn Tg mice could be the result of the increased phagocytosis capacity and enhanced Ag-presenting ability of macrophages and dendritic cells overexpressing the bovine FcRn transgene. As shown, the transgenic bFcRn is strongly expressed in macrophages and dendritic cells (**Figure 1A and 1B**), resulting in the enhanced processing of phagocytosed ICs and more effective loading of MHC II molecules. The strong expression of the bFcRn transgene in these two cell types is consistent with the expression of its mouse counterpart, i.e. mFcRn expression is similarly highly expressed in macrophages and dendritic cells as it has been shown by gene expression analysis performed by others (Immunological Genome Project; www.immgen.org). This suggests that the regulatory sequences of the bFcRn α-chain gene are properly regulated by the endogenous transcriptional machinery of the mouse. The higher expression ratio of the bFcRn to mFcRn in the bone marrow derived dendritic cells suggests that the bFcRn expression is strongly stimulated by IL-4 and/or GM-CSF, while these factors may not induce such a strong influence for the mFcRn expression. We currently analyze the transcriptional regulation of the bFcRn α-chain to further elucidate this exciting observation. Although previous work of others and our own study indicated FcRn expression in neutrophil granulocytes [13], [18] and B cells [15] we did not detect bFcRn expression at protein level in Western blot (**Figure 1A**). Perhaps a more sensitive method (confocal microscopy, FACS) would detect this protein in these cell types.

Our study revealed that the macrophages and dendritic cells from Tg animals have a significant increase in their phagocytic activity (4-fold in dendritic cells) (**Figure 2A–C**) and induce 6-fold higher proliferation of CD4^+^ T cells (**Figure 2D**). Since we did not detect any difference in the cell surface expression of the FcgRs, MHC class II, B7.1 and B7.2 between Tg and wt dendritic cells, the increased T cell proliferation was probably the result of the greater proportion of MHC class II loaded with OVA derived peptides in case of bFcRn Tg cells. The enhanced activation of T helper cells may also contribute to the elevated secondary humoral response and the increased diversity of Abs observed in immunized bFcRn Tg mice.

It is possible that the more efficient Ag presentation in bFcRn Tg mice (**Figure 2D**) results in a more robust activation of the existing Ag-specific memory B cells, which would explain the higher levels of Ag-specific IgG in the bFcRn Tg mice observed previously [18], [20] and also in this study (**Figure 4** **,** **6**). In addition, a recent report demonstrated that the ICs formed between Ags and pre-existing Abs from the primary immune response activate naïve B cells, inducing them to respond with accelerated kinetics and increased magnitude during the secondary immune response [40], [41]. In bFcRn Tg mice, overexpression of bovine FcRn leads to an increase in the half-life of IgG, which remain in the circulation and form ICs upon secondary exposure to the Ag. These ICs induce a higher proliferation of Ag-specific memory B cells and the activation of naïve B cells, resulting in a synergistic enhancement of the secondary humoral immune response.

Encouraged by experimental results that consistently demonstrate a superior immune response capability in FcRn overexpressing animals; we investigated the diversity of OVA induced Abs in Tg and wt mice using microarray-based oligopeptide scanning. The microarrays used in our study contained a library of overlapping peptides covering the whole sequence of OVA, similarly to a previously described method [42]. Although the majority of Ag-Ab interactions are based on conformational epitopes and oligopeptide scanning only allows for the identification of linear epitopes, this method is best suited to map epitopes from a large population of Abs generated against a defined target. We observed that the number of epitopes recognized by IgM is substantially increased in bFcRn Tg mice (**Figure 5**), suggesting that a significantly greater number of naïve B cells are activated in Tg mice as compared to the wt controls. We also detected significantly higher titers of peptide-specific IgG in Tg mice, however the epitopes targeted by IgG are similar between the two groups. Since our previous experiments demonstrated that the augmented immune response in the Tg mice results in larger numbers of Ag-specific B cells [18], [19], [20], we propose that a larger number of memory B cells is activated in these Tg mice in case of at least some of the recognized epitopes. Whether the more B cells recognizing a given peptide originate from more clones leading to greater diversity in Tg animals remains to be determined (**Figure 6**).

It is well-known that the immune system, with its ability to recognize a wide array of B- and T cell epitopes, can exhibit a strong preference for a limited set of epitopes. An earlier study showed that immunization with oligopeptides generates a multi-specific IgM response, whereas the mature IgG response was found to focus primarily on one tetrapeptide sequence (immunodominant epitope) [43]. Interestingly, the selection for mono-specificity occurs at or around the time of IgM to IgG class switch and is only reflected in the IgG population. Yet, when immunodominant epitopes are removed from the Ag, IgG are produced against other potentially antigenic determinants [43]. Thus, it is possible to refocus antibody response by targeted dampening of an immunodominant epitope [44], [45], [46]. This suggests that immunodominant epitopes can block effective immune response to subdominant epitopes even when the immune response is significantly augmented as in the case of the bFcRn Tg mice.

Whereas the enhancement of epitope recognition in FcRn-overexpressing animals is most compelling at the IgM level, the translation from IgM to IgG appears to be suppressed by immunodominant epitopes present on OVA. However, the observation of the much augmented immune response displayed by FcRn Tg mice is consistent with our findings of significantly elevated IgG quantities in this paper (approximately 3-fold higher titers of IgG in FcRn Tg mice than in wt). These results are also in line with our previous reports describing a substantial increase in the levels of Ag-specific IgG in FcRn Tg mice immunized with Ags that are weakly immunogenic in wt mice and, therefore, not affected by immunodominance, including a conserved influenza hemagglutinin (HA) peptide [20] and human CXCR4 [29]. Experiments with a series of other Ags, which cannot induce a meaningful immune response in standard models, are currently being done by our group and by other independent parties. To the best of our knowledge no other method has been described to date which shows a similar capability to augment the immune response both by qualitative as well as by quantitative measures.

## Supporting Information

Figure S1 **Sequences of peptides used for OVA epitope mapping.** BX indicates N-terminal biotinylation of each peptide. The biotinylated peptides were mixed with streptavidin and the complexes were then printed on the microchips for analyses.
(DOC)
