# Supplementary material for: FcRn Overexpression in Transgenic Mice Results in Augmented APC Activity and Robust Immune Response with Increased Diversity of Induced Antibodies
Source: PLoS One. 2012 Apr 30;7(4):e36286. doi: 10.1371/journal.pone.0036286 (PMC3340356; doi:10.1371/journal.pone.0036286)
Supplement: Figure S1 — Sequences of peptides used for OVA epitope mapping. BX indicates N-terminal biotinylation of each peptide. The biotinylated peptides were mixed with streptavidin and the complexes were then printed on the microchips for analyses. (DOC) [file pone.0036286.s001.doc]

**Figure S1.**

| 1 | BXMGSIGAASMEFC | 25 | BXVYSFSLASRLYA | 49 | BXDTQAMPFRVTEQ | 73 | BXEEKYNLTSVLMA |
| --- | --- | --- | --- | --- | --- | --- | --- |
| 2 | BXGAASMEFCFDVF | 26 | BXSLASRLYAEERY | 50 | BXMPFRVTEQESKP | 74 | BXNLTSVLMAMGIT |
| 3 | BXMEFCFDVFKELK | 27 | BXRLYAEERYPILP | 51 | BXVTEQESKPVQMM | 75 | BXVLMAMGITDVFS |
| 4 | BXFDVFKELKVHHA | 28 | BXEERYPILPEYLQ | 52 | BXESKPVQMMYQIG | 76 | BXMGITDVFSSSAN |
| 5 | BXKELKVHHANENI | 29 | BXPILPEYLQCVKE | 53 | BXVQMMYQIGLFRV | 77 | BXDVFSSSANLSGI |
| 6 | BXVHHANENIFYCP | 30 | BXEYLQCVKELYRG | 54 | BXYQIGLFRVASMA | 78 | BXSSANLSGISSAE |
| 7 | BXNENIFYCPIAIM | 31 | BXCVKELYRGGLEP | 55 | BXLFRVASMASEKM | 79 | BXLSGISSAESLKI |
| 8 | BXFYCPIAIMSALA | 32 | BXLYRGGLEPINFQ | 56 | BXASMASEKMKILE | 80 | BXSSAESLKISQAV |
| 9 | BXIAIMSALAMVYL | 33 | BXGLEPINFQTAAD | 57 | BXSEKMKILELPFA | 81 | BXSLKISQAVHAAH |
| 10 | BXSALAMVYLGAKD | 34 | BXINFQTAADQARE | 58 | BXKILELPFASGTM | 82 | BXSQAVHAAHAEIN |
| 11 | BXMVYLGAKDSTRT | 35 | BXTAADQARELINS | 59 | BXLPFASGTMSMLV | 83 | BXHAAHAEINEAGR |
| 12 | BXGAKDSTRTQINK | 36 | BXQARELINSWVES | 60 | BXSGTMSMLVLLPD | 84 | BXAEINEAGREVVG |
| 13 | BXSTRTQINKVVRF | 37 | BXLINSWVESQTNG | 61 | BXSMLVLLPDEVSG | 85 | BXEAGREVVGSAEA |
| 14 | BXQINKVVRFDKLP | 38 | BXWVESQTNGIIRN | 62 | BXLLPDEVSGLEQL | 86 | BXEVVGSAEAGVDA |
| 15 | BXVVRFDKLPGFGD | 39 | BXQTNGIIRNVLQP | 63 | BXEVSGLEQLESII | 87 | BXSAEAGVDAASVS |
| 16 | BXDKLPGFGDSIEA | 40 | BXIIRNVLQPSSVD | 64 | BXLEQLESIINFEK | 88 | BXGVDAASVSEEFR |
| 17 | BXGFGDSIEAQCGT | 41 | BXVLQPSSVDSQTA | 65 | BXESIINFEKLTEW | 89 | BXASVSEEFRADHP |
| 18 | BXSIEAQCGTSVNV | 42 | BXSSVDSQTAMVLV | 66 | BXNFEKLTEWTSSN | 90 | BXEEFRADHPFLFC |
| 19 | BXQCGTSVNVHSSL | 43 | BXSQTAMVLVNAIV | 67 | BXLTEWTSSNVMEE | 91 | BXADHPFLFCIKHI |
| 20 | BXSVNVHSSLRDIL | 44 | BXMVLVNAIVFKGL | 68 | BXTSSNVMEERKIK | 92 | BXFLFCIKHIATNA |
| 21 | BXHSSLRDILNQIT | 45 | BXNAIVFKGLWEKT | 69 | BXVMEERKIKVYLP | 93 | BXIKHIATNAVLFF |
| 22 | BXRDILNQITKPND | 46 | BXFKGLWEKTFKDE | 70 | BXRKIKVYLPRMKM | 94 | BXATNAVLFFGRCV |
| 23 | BXNQITKPNDVYSF | 47 | BXWEKTFKDEDTQA | 71 | BXVYLPRMKMEEKY | 95 | BXNAVLFFGRCVSP |
| 24 | BXKPNDVYSFSLAS | 48 | BXFKDEDTQAMPFR | 72 | BXRMKMEEKYNLTS |  |  |
